# Efficacy and Safety Assessment of the Addition of Bevacizumab to Adjuvant Therapy Agents in Cancer Patients: A Systematic Review and Meta-Analysis of Randomized Controlled Trials

**DOI:** 10.1371/journal.pone.0136324

**Published:** 2015-09-02

**Authors:** Fariba Ahmadizar, N. Charlotte Onland-Moret, Anthonius de Boer, Geoffrey Liu, Anke H. Maitland-van der Zee

**Affiliations:** 1 Division of Pharmacoepidemiology and Clinical Pharmacology, Utrecht Institute for Pharmaceutical Sciences (UIPS), Utrecht University, Utrecht, the Netherlands; 2 Julius Center for Health Sciences and Primary Care, UMC Utrecht, the Netherlands; 3 Division of Medical Oncology and Hematology, Department of Medicine, Princess Margaret Hospital/University Health Network and University of Toronto, Toronto, Ontario, Canada; 4 Division of Epidemiology, Dalla Lana School of Public Health, Toronto, Ontario, Canada; German Cancer Research Center (DKFZ), GERMANY

## Abstract

**Aim:**

To evaluate the efficacy and safety of bevacizumab in the adjuvant cancer therapy setting within different subset of patients.

**Methods & Design/ Results:**

PubMed, EMBASE, Cochrane and Clinical trials.gov databases were searched for English language studies of randomized controlled trials comparing bevacizumab and adjuvant therapy with adjuvant therapy alone published from January 1966 to 7^th^ of May 2014. Progression free survival, overall survival, overall response rate, safety and quality of life were analyzed using random- or fixed-effects models according to the PRISMA guidelines. We obtained data from 44 randomized controlled trials (30,828 patients). Combining bevacizumab with different adjuvant therapies resulted in significant improvement of progression free survival (log hazard ratio, 0.87; 95% confidence interval (CI), 0.84–0.89), overall survival (log hazard ratio, 0.96; 95% CI, 0.94–0.98) and overall response rate (relative risk, 1.46; 95% CI: 1.33–1.59) compared to adjuvant therapy alone in all studied tumor types. In subgroup analyses, there were no interactions of bevacizumab with baseline characteristics on progression free survival and overall survival, while overall response rate was influenced by tumor type and bevacizumab dose (p-value: 0.02). Although bevacizumab use resulted in additional expected adverse drug reactions except anemia and fatigue, it was not associated with a significant decline in quality of life. There was a trend towards a higher risk of several side effects in patients treated by high-dose bevacizumab compared to the low-dose e.g. all grade proteinuria (9.24; 95% CI: 6.60–12.94 vs. 2.64; 95% CI: 1.29–5.40).

**Conclusions:**

Combining bevacizumab with different adjuvant therapies provides a survival benefit across all major subsets of patients, including by tumor type, type of adjuvant therapy, and duration and dose of bevacizumab therapy. Though bevacizumab was associated with increased risks of some adverse drug reactions such as hypertension and bleeding, anemia and fatigue were improved by the addition of bevacizumab.

## Introduction

Bevacizumab (BV), a humanized recombinant monoclonal antibody against vascular endothelial growth factor (VEGF), was approved by the US food and drug administration (FDA) on the market based on its effectiveness in metastatic cancers. Bevacizumab specifically binds to the VEGF-A protein, thereby inhibiting the process of angiogenesis.

Many randomized controlled trials (RCTs) and several meta-analyses on the efficacy and safety of BV in different tumor types have been published. From these studies, while BV added to chemotherapy improved progression free survival (PFS) and overall survival (OS), there was no significant influence on quality of life (QOL) but there were increased risks of serious adverse drug reactions (ADRs). There was controversy on the dose-effect relations of BV and ADRs: while some studies found increased risks of the occurrence of some ADRs e.g. all grade hypertension (RR: 7.5, 95% CI: 4.2–13.4 vs. RR: 3.0, 95%CI: 2.2–4.2), and high-grade bleeding (RR: 3.02, 95% CI:1.85–4.95 vs. RR: 1.27,95%CI: 0.95–1.7) [1,2] for the high-dose BV compared to low-dose, whereas a recent safety meta-analysis of 13 heterogeneous trials did not [3]. However, defining which of any benefited more or less from BV has not been extensively studied. Therefore, and because there have been new RCTs published after the latest published meta-analysis [4], we conducted a large meta-analysis to examine predictive factors for BV efficacy and safety by performing a series of subgroup, meta-regression and sensitivity analyses. In addition, we systematically assessed heterogeneity and publication bias.

## Methods

### Data source

All published RCTs on the efficacy and safety of BV in different tumor types were collected by conducting a literature search using PubMed, EMBASE, Cochrane and Clinical trials.gov database with the keywords shown in S1 Table **(See Appendix)**. Furthermore, we searched abstracts and virtual meeting presentations from websites of the American Society of Clinical Oncology (ASCO), European Society for Medical Oncology (ESMO), Federation of European Cancer Societies (FECS) and San Antonio Breast Cancer Symposium (SABCS) to identify relevant RCTs. Further information was retrieved through a manual search of references from recent meta-analyses and relevant published trials.

### Inclusion and exclusion criteria

All phase 2 or 3 RCTs were included in our study if there was a direct comparison between BV in combination with adjuvant therapy and adjuvant therapy alone available (experimental arm: BV plus adjuvant therapy agent (s); control arm: adjuvant therapy with or without placebo) in patients with metastatic cancers. Only publications in English language and from January 1966 to 7^th^ of May 2014 were considered. Trials in pediatric populations and trials where BV was used for the treatment of macular retinopathy and brain tumors were excluded from this meta-analysis because of different outcome measurements. Two investigators (FA and AdB) independently applied the inclusion and exclusion criteria to select the relevant trials.

### Data extraction and clinical end points

Data extraction was conducted in agreement with the Preferred Reporting Items for Systematic Reviews and Meta-Analyses (PRISMA) guidance (**S1 PRISMA Checklist**) [5]. The clinical end points used for this study were PFS, defined as the time from random assignment to first reported progression or all-cause mortality in the absence of previously documented tumor progression, OS, defined as the time from random assignment to death which can be from any cause, censoring patients who had alive at the date last visit, overall response rate (ORR), defined as the sum of partial and complete response rates (according to the Response Evaluation Criteria in Solid Tumors) [6], ADRs were graded according to the Common Toxicity Criteria version 3 (http://ctep.cancer.gov) and QOL assessed at baseline and during the follow-up time until disease progression.

Hazard Ratios (HRs) for PFS and OS, median PFS (mPFS) and median OS (mOS), the number of patients with ORR and the number of adverse drug reactions (ADRs) were extracted from the papers. Furthermore, the first author, year of publication, trial design characteristics (study phase, outcome measures, tumor type, therapy regime for each arm, dose of treatment, median time of follow-up, median duration of BV therapy and time points of response assessment), patient characteristics (median age and number of patients evaluated for efficacy and safety in each arm) were extracted. In case of missing data for HR as a point estimate in trials, authors were contacted via email to provide the necessary information.

### Risk of bias assessment

Quality assessment of the publications included was performed independently by three investigators using the Cochrane Collaboration’s tool (http://handbook.cochrane.org/chapter_8/8_assessing_risk_of_bias_in_included_studies.htm). This means that the trials were rated for domains of random sequence generation, allocation concealment, blinding of participants and personnel, blinding of primary outcomes (PFS, OS, ORR) assessment, blinding of secondary outcomes (safety and QOL) assessment, incomplete PFS, OS and ORR data, incomplete safety data, selective reporting and other biases. In the case of any disagreement between investigators to rate the quality of each trial consensus was reached. When there was insufficient information to permit the evaluation of the quality it was rated as unclear (uncertain risk of bias).

### Data analysis

Overall pooled estimates, together with 95% confidence intervals (CI) of the PFS, OS, ORR and safety outcomes were obtained using either a fixed-effects model or in the event of heterogeneity, a random-effects model. Relative risks (RRs) and 95% CIs were calculated to assess the ORR and safety of BV compared with control group. Subgroup analyses to identify the overall impact of patient and trial characteristics on BV efficacy were performed for the following characteristics: different tumor types, BV dose (high-dose (5 mg/kg weekly) and low-dose (2.5 mg/kg weekly)), types of adjuvant therapies (platinum (cisplatin, carboplatin, or oxaliplatin) and taxanes (paclitaxel or docetaxel) versus non-platinum (non-platinum and nontaxane-based)), age of participants (50–55, 56–60, 61–66, >66 years old), median duration of follow-up (6–12, 13–24, 25–36, >37 months), median duration of BV therapy (<12, 12–24, 25–36, >37 weeks) and timing of response assessment (6, 8–12, 24 weeks). These subgroup analyses were performed for all trials combined, colorectal cancer, non-small cell lung cancer (NSCLC) and breast cancer patients separately. The time interval between mOS and mPFS was measured as survival post progression (SPP) in different tumor types including colorectal cancer, NSCLC, breast cancer and ovarian cancer. The validation of PFS as a surrogate endpoint for OS in all combined trials was tested using the Spearman’s rank correlation. Linearity between the log HRs of PFS and OS was also assessed in a linear regression model. The correlation between PFS and OS was further studied in subgroup analyses in different tumor types including colorectal cancer, NSCLC and breast cancer. Publication bias was evaluated by using funnel plots and the Egger test was applied to measure any asymmetry. Heterogeneity of the studies was tested by the I^2^ measure of inconsistency with 25% corresponding to low heterogeneity, 50% to moderate and 75% to high. If heterogeneities existed, one of the following techniques was used to explain them: random-effects models for meta-analysis, subgroup analyses, or meta-regression analyses. Meta-regression analyses were also performed for the BV dose, median age of participants, median duration of follow-up and median duration of BV therapy. To evaluate the relation between BV doses and the risk of ADRs a subgroup analysis was performed. In addition, sensitivity analysis was applied by omitting one study in each turn and investigated the influence of a single study on the overall meta-analysis estimate [7] when necessary. All statistical analyses were conducted using STATA 10/SE (StataCorp. 2007. Stata Statistical Software: Release 10. College Station, TX: StataCorp LP).

## Results

### Search results

**Fig 1:**
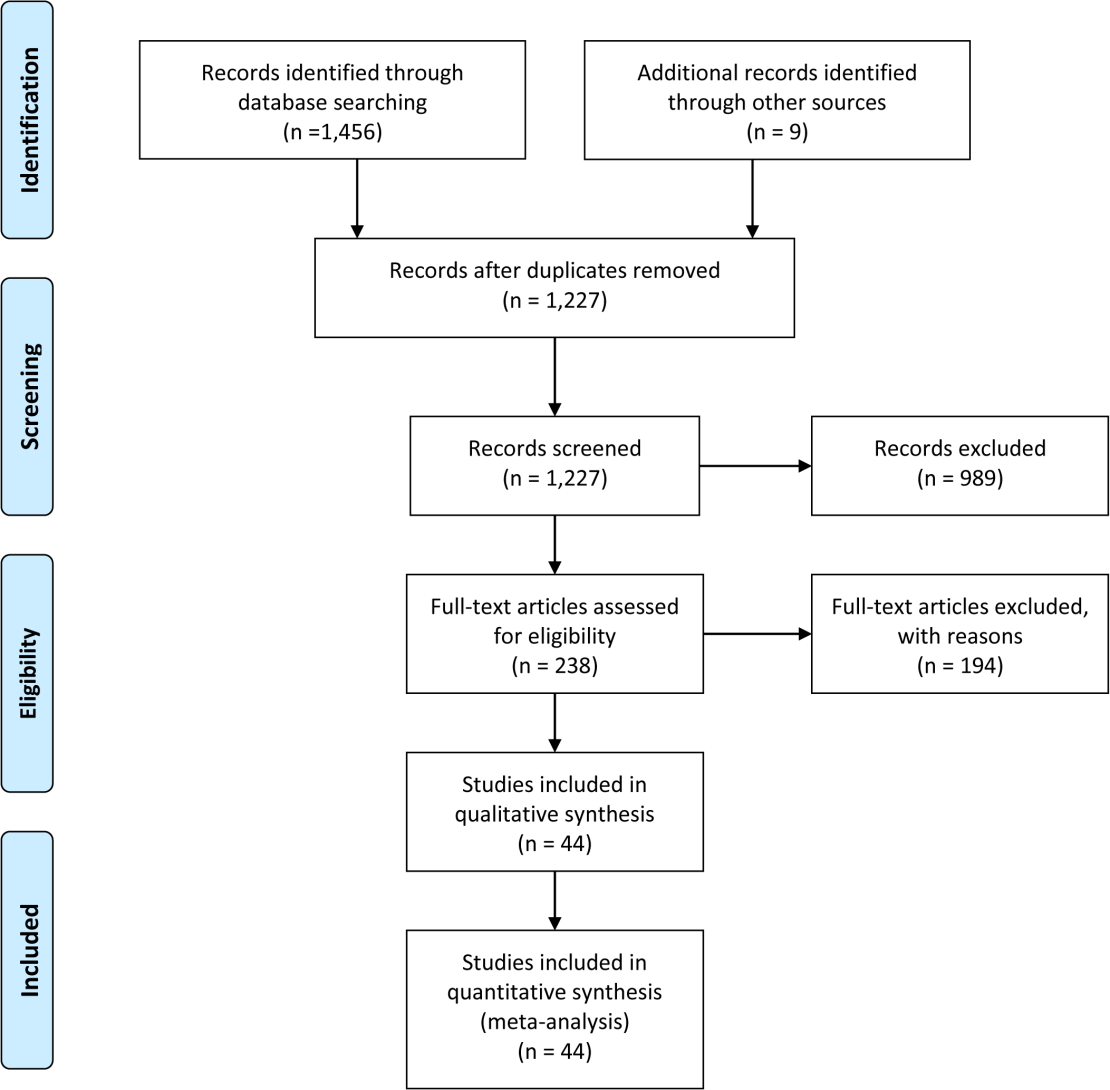
A Flow Diagram showing the RCTs selection.

**Fig 1** shows a flow chart for the selection procedure of the trials. Our literature search yielded 1,465 published articles on BV safety and efficacy and after applying the inclusion and exclusion criteria a total of 44 RCTs [8–11,11–19,19–28,29–51] were selected for the meta-analyses **(Fig 1)**.

### Study characteristics

The characteristics of the included trials are summarized in **Table 1**. A total of 30,828 patients (BV, n = 16,266; control, n = 14,562) from 44 RCTs were included in the meta-analysis. Underlying malignancies were included colorectal cancer (13 studies), breast cancer (10 studies), NSCLC (7 studies), ovarian cancer (4 studies), renal cell cancer (2 studies), pancreatic cancer (2 studies), gastric cancer (1 study), melanoma cancer (1 study), prostate cancer (1 study), mesothelioma cancer (1 study), cervical cancer (1 study) and Follicular lymphoma (1 study). Five trials evaluated BV in different arms, either different doses of BV [14,26,27,35] or its combination with different adjuvant therapy agents [34] with a control group. Sample sizes ranged from 23 to 2,867 patients, with 24 trials including more than 500 patients each. In all trials, patients were randomly assigned to either the control or the BV group. Eleven (25%) trials were phase II and 33 (75%) were phase III studies. Trial treatment regimens varied by tumor types and the BV dose ranged from 2.5 to 5 mg/kg weekly. The median age of patients in all trials combined was almost 59 years in both groups. All studies recruited both male and female participants (except trials of metastatic breast cancer, ovarian cancer, prostate cancer and cervical cancer) however outcomes were not reported stratified by gender.

**Table 1:** Characteristics of trials included in the meta-analysis.

RCTs	Tumor type	Allocation sequences	Trial phase	Enrolled patients	Adjuvant therapy	BV dose, mg/kg/week	Median duration of follow–up (months)	Time points of response assessment (weeks)
**Bennouna, J et al, 2013**	MCRC	C	III	820	Oxaliplatin plus Irrinotecan	2.5	11.1	NA
**Cunnigham, D et al, 2013**	MCRC	C	III	280	Capecitabine	2.5	24.8	9
**De Gramont, A et al, 2012**	MCRC	C	III	2867	Oxaliplatin, fluorouracil, and leucovorin (FOLFOX4)	2.5	48	24
**Dotan, E et al, 2012**	MCRC	C	II	23	Capecitabine plus Oxaliplatin plus Cetuximab	2.5	25.9	6
**Kemeny, NE et al, 2011**	MCRC	C	II	73	Oxaliplatine plus Fluorouracil plus Leucovorin	2.5	30	NA
**Guan, ZZ et al, 2011**	MCRC	C	III	214	Irinotecan plus leucovorin, and 5-fluorouracil	2.5	NA	6
**Allegra,C J et al, 2011**	MCRC	C	III	2710	FOLFOX6	2.5	35.6	NA
**Tebbutt, N et al, 2009**	MCRC	C	III	471	Capecitabine	2.5	31	6
**Stathopulos, GP et al, 2010**	MCRC	C	III	222	leucovorin plus 5-fluorouracil plus irinotecan	2.5	36	8
**Saltz, LB et al, 2008**	MCRC	B	III	1401	FOLFOX-4 / XELOX	2.5	27.6	6
**Giantanio, BJ et al, 2007**	MCRC	C	III	829	FOLFOX4	5	28	12
**Hurwitz, HI et al, 2004**	MCRC	B	III	813	Irinotecan plus fluorouracil, and leucovorin	2.5	18	6
**Kabbinavar, F et al, 2003**	MCRC	C	II	104	Fluorouracil l/ leucovorin	2.5, 5	17.6	8
**Cameron, D et al, 2013**	MBC	C	III	2591	Antracycline plus Taxane	5	31.5	NA
**Luca, G et al, 2013**	MBC	C	III	424	Docetaxel plus Trastuzumab	5	26	9
**Von Minckwitz, G et al, 2012**	MBC	C	III	1948	Epirubicin plus cyclophosphamide plus docetaxel	5	NA	6
**Bear, H et al, 2012**	MBC	C	III	1206	Docetaxel plus Capecitabine	5	NA	NA
**Brufsky, AM et al, 2011**	MBC	A	III	684	Capecitabine plusa Taxane	5	15	6
**Robert, NJ et al, 2011**	MBC	A	III	618	Capecitabine plus Taxane–based	5	15.6, 19.2	9
**Martin, M et al, 2011**	MBC	A	II	191	Paclitaxel	2.5	NA	8
**Miles, DW et al, 2010**	MBC	A	III	736	Docetaxel	2.5, 5	25	9
**Miller, K et al, 2007**	MBC	C	III	722	Paclitaxel	5	41.6	12
**Miller, KD et al, 2005**	MBC	C	III	462	Capecitabine	5	14.8	6
**Niho, S et al, 2012**	NSCLC	C	II	180	Carboplatin plus Paclitaxel	5	NA	6
**Herbst, RS et al, 2011**	NSCLC	C	III	636	Erlotinib	5	19	6
**Spigel, DR et al, 2011**	NSCLC	A	II	102	Cisplatin or Carboplatin plus Etoposide	5	7.8	6
**Reck, M et al, 2009**	NSCLC	B	III	1043	Cisplatin plus Gemcitabine	2.5, 5	7	9
**Herbst,R et al, 2007**	NSCLC	C	II	120	Docetaxel or Pemetrexed	5	15.8	6
**Sandler, A et al, 2006**	NSCLC	C	III	878	Paclitaxel plus Carboplatin	5	19	6
**Johnson, DH et al, 2004**	NSCLC	C	II	99	Carboplatin plus Paclitaxel	2.5, 5	14.7	6
**Eric, PL et al, 2014**	OC	C	III	361	Doxorubicin plus Paclitaxel plus Topotecan	5	13.9	8
**Aghajanian, C et al, 2012**	OC	A	III	484	Carboplatin plus Gemcitabine	5	24	9
**Perren,T et al, 2011**	OC	C	III	1528	Carboplatin plus Paclitaxel	2.5	28	6
**Burger, RA et al, 2011**	OC	A	III	1873	Paclitaxel plus Carboplatin	5	17.4	NA
**Kindler, HL et al, 2010**	MPC	A	III	602	Gemcitabine	2.5	11.3	6
**Cutsem, E et al, 2009**	MPC	A	III	607	Gemcitabine plus Erlotinib	2.5	6.7	8
**Rini, BI et al, 2010**	RCC	C	III	732	Interferon alpha	5	46.2	12
**Escudier, B et al, 2007**	RCC	A	III	649	Interferon alfa-2a	5	13.3	8
**Krishnansu, ST et al, 2014**	CC	C	III	452	Cisplatin plus Paclitaxel	5	20.8	NA
**John, DH et al, 2014**	RFL	C	II	60	Rituximab	5	34	NA
**Kim, KB et al, 2012**	MC	A	II	214	Carboplatin plus paclitaxel	5	13	6
**Kelly, WK et al, 2012**	PC	A	III	1050	Docetaxel plus prednisone	5	24	12
**Kindler, HL et al, 2012**	AM	A	II	115	Gemcitabine plus Cisplatin	5	NA	6
**Ohtsu, A et al, 2011**	GC	A	III	774	Cisplatin plus Capecitabine	2.5	11.4	6

**Abbreviations:** RCTs: randomized control trials; BV: bevacizumab; MCRC: metastatic colorectal cancer; GC: gastric cancer; MPC: metastatic pancreatic cancer; RCC: renal carcinoma cancer; NSCLC: non-small-cell lung carcinoma; MBC: metastatic breast cancer; AM: advanced mesothelioma; PC: prostate cancer; MC: melanoma carcinoma; OC: ovarian cancer; CC: cervical cancer; RFL: Relapsed Follicular Lymphoma, A: Double-blinded- placebo and active treatment control, B: Placebo and active treatment control, C: Active treatment control.

### Risk of bias assessment in all trials combined

Randomized treatment allocation sequences were generated in 44 RCTs. Fourteen trials were double blinded with placebo and active treatment controls, 3 trials had placebo and active treatment controls and the rest of trials had active treatment controls. **S2 Table** presents the risk of bias judgments for the 44 RCTs. According to our methodological assessment, the results showed a low risk of bias in most domains except for blinding across all outcomes; therefore, the overall quality of all trials combined was acceptable.

### Efficacy analyses in all trials combined

**Fig 2:**
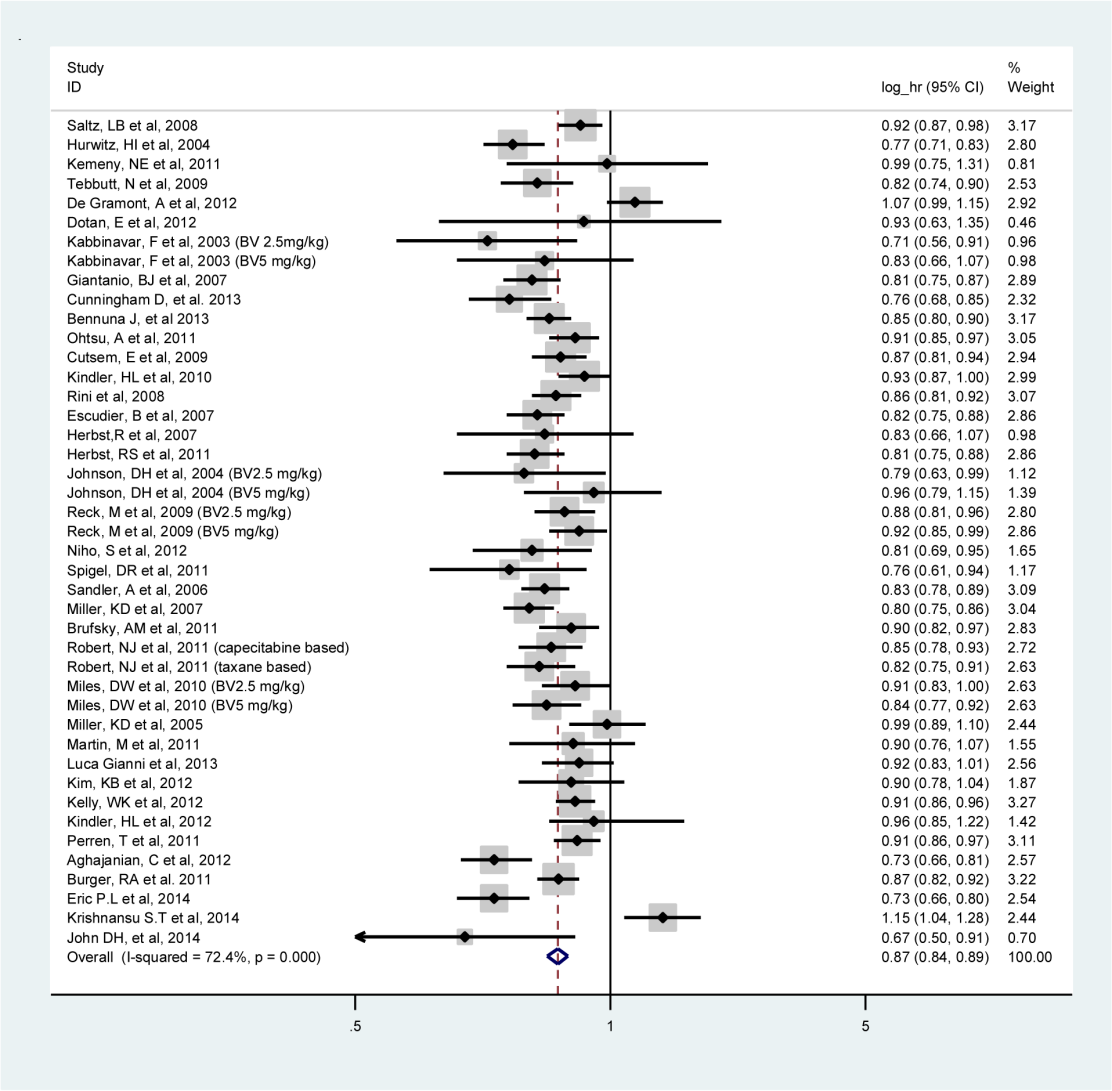
Meta-analysis of the log hazard ratios of progression free survival comparing bevacizumab and standard therapy in all trials combined.

The meta-analysis of PFS was based on 38 RCTs (**Table 2**). Combining BV with different adjuvant therapies resulted in a 13% risk reduction of PFS events (log HR, 0.87; 95% CI, 0.84–0.89; I^2^:72.4%, random-effects model) (**Fig 2**) with high heterogeneity attributed to the colorectal cancer (I^2^: 82.3%) and ovarian cancer (I^2^: 92.8%) trials. PFS statistically significantly improved in patients for all types of tumors except for patients with melanoma, mesothelioma and cervical cancers. No statistically significant differences between logs HRs of PFS were observed between the different tumor types.

**Table 2:** BV efficacy assessment in trials included in the meta-analysis.

	PFS					OS					ORR				
	No. of studies	No. of patients in BV group/sample size	No. of patients in control group/sample size	Log HR (95% CI)	I^2^	No. of studies	No. of patients in BV group/sample size	No. of patients in control group/sample size	Log HR (95% CI)	I^2^	No. of studies	No. of patients in BV group/sample size	No. of patients in control group/sample size	RR (95% CI)	I^2^
**All trial combined**	**38**	**12221/24696**	**10756/24696**	0.87 (0.84–0.89)	72.4	**34**	**11611/23821**	**10491/23821**	0.96 (0.94–0.98)	22.2	**24**	**7898/14596**	**6385/14596**	**1.46 (1.33–1.59)**	**71.3**
Colorectal cancer	10	3364/7681	3346/7681	0.85 (0.79–0.92)	82.3	9	3224/7401	3206/7401	0.93 (0.87–0.99)	61.1	6	1606/3384	1514/3384	1.40 (1.01–1.95)	84.2
NSCLC	7	1712/3058	1279/3058	0.85 (0.82–0.89)	13.0	6	1016/2015	932/2015	0.94 (0.90–0.98)	0	4	1188/1961	773/1961	1.60 (1.37–1.87)	9.4
Breast cancer	7	2670/4456	1737/4456	0.87 (0.84–0.91)	50.5	6	3658/6432	2725/6432	0.98 (0.94–1.01)	0	6	2454/4032	1529/4032	1.36 (1.25–1.47)	10.4
Pancreatic cancer	2	608/1209	601/1209	0.90 (0.85–0.96)	36.5	2	608/1209	601/1209	1.01 (0.94–1.08)	0	2	608/1209	601/1209	1.41 (1.02–1.95)	0
Ovarian cancer	4	2035/4698	2038/4698	0.86 (0.76–0.99)	92.8	3	1044/2718	1049/2718	0.97 (0.91–1.03)	0	3	1185/2373	1188/2373	1.47 (1.25–1.73)	68.7
Renal Cancer	2	696/1381	685/1381	0.84 (0.80–0.89)	0	2	696/1381	685/1381	0.95 (0.90–0.99)	0	1	1016/2015	932/2015	**2.55 (1.81–3.61)**	**-**
Gastric Cancer	1	387/774	387/774	0.91 (0.85–0.97)	-	1	387/774	387/774	0.94 (0.87–1.01)	-	1	387/774	387/774	1.29 (1.05–1.58)	-
Prostate Cancer	1	524/1050	526/1050	0.91 (0.86–0.96)	-	1	524/1050	526/1050	0.96 (0.90–1.02)	-	-	-	-	-	-
Melanoma Cancer	1	143/214	71/214	0.90 (0.78–1.04)	-	1	143/214	71/214	0.84 (0.71–0.99)	-	1	143/214	71/214	1.49 (0.83–2.68)	-
Mesothelioma Cancer	1	53/115	55/115	0.96 (0.79–1.15)	-	1	53/115	55/115	1.02 (0.85–1.22)	-	-	-	-	-	-
Cervical cancer	1	227/452	225/452	1.15 (1.04–1.28)	-	1	227/452	225/452	1.08 (0.92–1.28)	-	-	-	-	-	-
Lymphoma	1	29/60	31/60	0.67 (0.50–0.91)	-	1	29/60	31/60	0.67 (0.44–1.02)	-	-	-	-	-	-

**Abbreviations:** PFS: progression free survival; OS: overall survival; ORR: overall response rate; HR: hazard ratio; RR: relative risk; BV: bevacizumab; NSCLC: non-small cell lung cancer.

**Fig 3:**
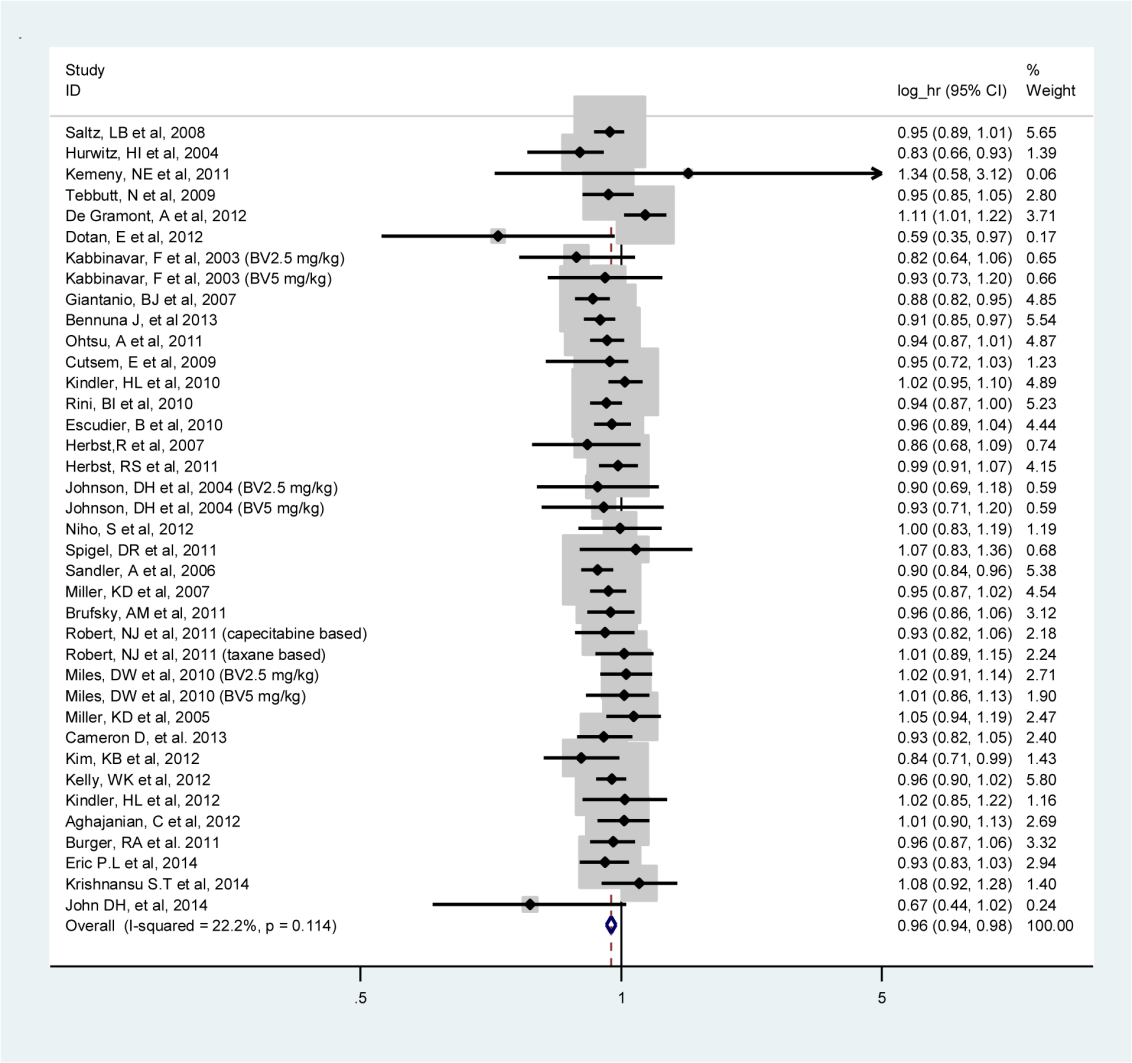
Meta-analysis of the logs hazard ratios of overall survival comparing bevacizumab and standard therapy in all trials combined.

The meta-analysis of OS was based on 34 RCTs. Adding BV caused a 4% risk reduction of OS events as compared with regimens without BV (log HR, 0.96; 95% CI, 0.94–0.98; I^2^:22.2%, fixed-effects model) (**Fig 3**). When investigating by type of tumor, OS was significantly improved only in colorectal cancer, NSCLC, renal cancer and melanoma cancer. Again the results showed no significant difference between logs HRs of OS in different tumor types.

**Fig 4:**
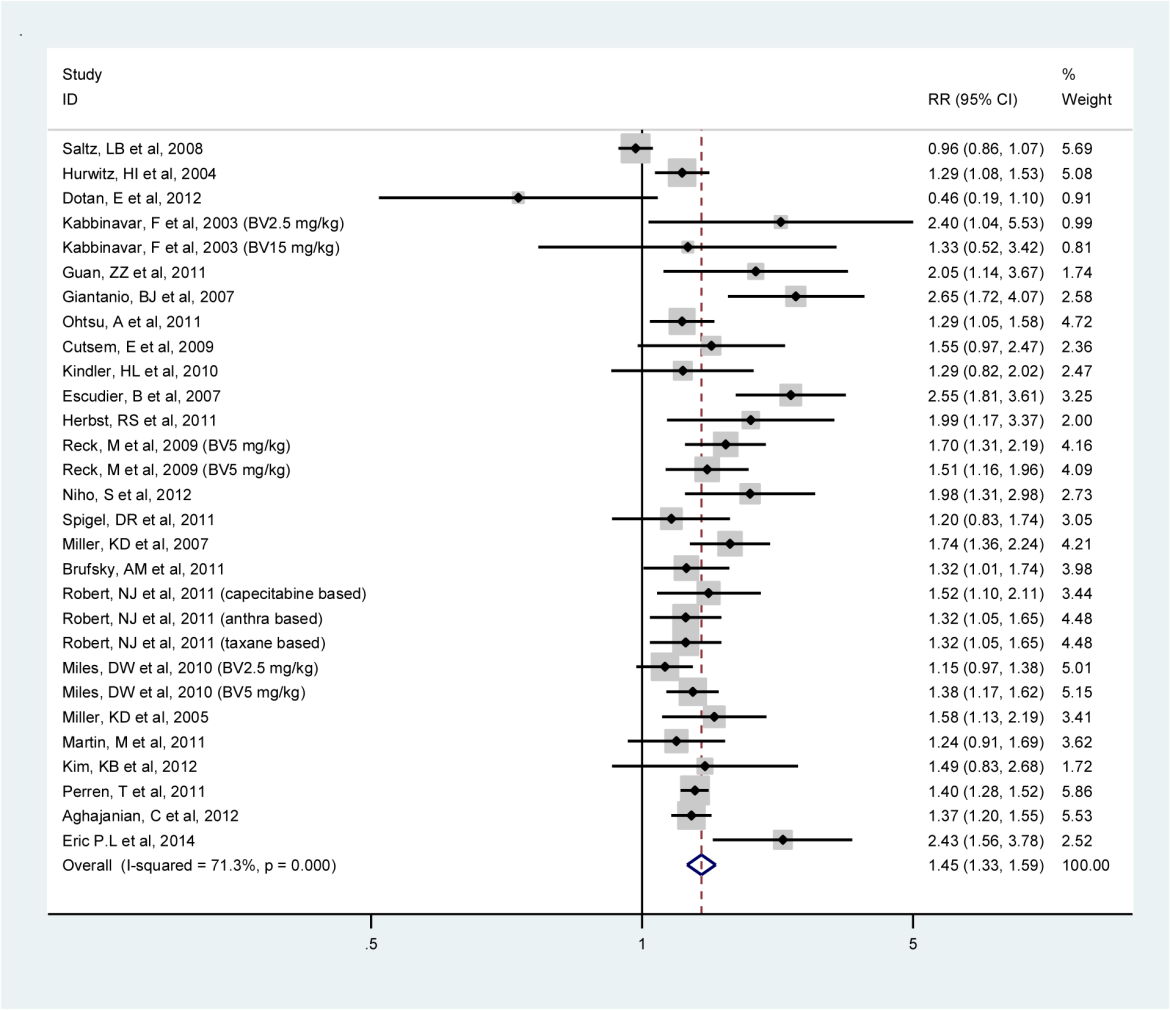
Meta-analysis of the risk ratios of overall response rate comparing bevacizumab and standard therapy in all trials combined.

The meta-analyzed RR of the ORR associated with the addition of BV to adjuvant therapy in 24 studies was 1.46 (95% CI: 1.33–1.59; I^2^: 71.3%, random-effects model) (**Fig 4**). High heterogeneity was observed in the colorectal cancer (I^2^: 84.2%) trials. The ORR was statistically significantly improved in all tumor types except in patients with melanoma cancer. Furthermore, the highest improvement of ORR was observed among renal cancer patients (RR, 2.55; 95% CI, 1.81–3.61). This was statistically significant different from the other tumor types.

### Efficacy subgroup analyses

As shown in Tables 3 and 4, patient and trial characteristics did not modify the effects of BV on PFS and OS. For specific tumor types including colorectal cancer, NSCLC and breast cancer, the overall pooled estimates of logs HRs of PFS (Figures A, B and C in **S1 File**) and OS (Figures A, B and C in **S2 File**) and RRs of ORR (Figures A, B and C in **S3 File**) comparing BV and standard chemotherapy are shown in forest plots.

**Table 3:** Efficacy subgroup analyses in all trials combined.

	PFS			OS			ORR		
	No. of studies	Log HR (95% CI)	I^2^	No. of studies	Log HR (95% CI)	I^2^	No. of studies	RR (95% CI)	I^2^
**BV dose**									
2.5 mg/kg	17	0.88 (0.84–0.92)	72.3	13	0.96 (0.92–1.01)	50.4	11	1.26 (1.09–1.45)	74.6
5 mg/kg	25	0.86 (0.83–0.89)	71.6	24	0.95 (0.93–0.97)	0	15	1.58 (1.43–1.74)	-
**Chemotherapy regimen**									
Platinum	30	0.88 (0.85–0.91)	74.5	25	0.96 (0.93–0.98)	27.5	17	1.40 (1.26–1.56)	77.0
Non-platinum	13	0.84 (0.80–0.88)	62.6	11	0.96 (0.93–1.00)	13.7	10	1.58 (1.36–1.84)	43.6
**Age (years)**									
50–55	8	0.88 (0.83–0.93)	55.6	6	0.98 (0.93–1.03)	0	7	1.32 (1.20–1.44)	0
56–60	14	0.87 (0.83–0.92)	80.5	11	0.96 (0.92–1.01)	52.4	12	1.34 (1.18–1.52)	77.9
61–65	10	0.84 (0.80–0.88)	58.2	10	0.95 (0.92–0.98)	15.9	7	**2.04 (1.67–2.49)**	33.1
>66	3	0.84 (0.75–0.95)	68.9	3	0.94 (0.87–1.02)	25.6	-		
**Median Follow-up (months)**									
6–12	6	0.88 (0.85–0.92)	26.1	5	0.96 (0.91–1.01)	26.5	5	1.40 (1.22–1.60)	0
13–24	18	0.84 (0.80–0.89)	79.7	17	0.95 (0.93–0.98)	7.6	11	1.52 (1.35–1.72)	53.8
25–36	9	0.88 (0.84–0.92)	47.5	8	0.93 (0.88–0.99)	37.7	6	1.32 (1.03–1.69)	90.4
>37	3	0.90 (0.77–1.07)	93.9	3	0.99 (0.90–1.09)	78.9	-	-	-
**Median duration of therapy (weeks)**									
<12	-	-	-	-	-	-	-	-	-
12–24	10	0.85 (0.79–0.92)	83.1	9	0.97 (0.91–1.03)	62.2	7	1.74 (1.41–2.16)	50.7
25–36	10	0.84 (0.80–0.89)	70.0	8	0.96 (0.92–0.98)	0	8	1.35 (1.14–1.60)	79.9
>37	4	0.83 (0.76–0.90)	61.1	3	0.91 (0.83–1.00)	23.3	2	1.78 (0.90–3.53)	92.0
**Time points of response assessment (weeks)**									
6	17	0.87 (0.84–0.91)	55.7	16	0.95 (0.92–0.98)	22.9	13	1.34 (1.16–1.54)	74.1
8–12	16	0.83 (0.80–0.87)	66.0	12	0.94 (0.92–0.97)	0	11	1.68 (1.41–1.99)	74.2
24	1	1.07 (0.99–1.16)	-	1	1.11 (1.01–1.22)	-	-	-	-

**Abbreviations:** PFS: progression free survival; OS: overall survival; ORR: overall response rate; HR: hazard ratio; RR: relative risk; BV: bevacizumab.

**Table 4:** Efficacy subgroup analyses in different tumor types.

	PFS			OS			ORR		
	N. of studies	Log HR (95% CI)	I^2^	N. of studies	Log HR (95% CI)	I^2^	N. of studies	RR (95% CI)	I^2^
**Metastatic Colorectal Cancer**									
**BV dose**									
2.5 mg/kg	9	0.86 (0.79–0.94)	84.8	8	0.94 (0.87–1.01)	65.1	5	1.22 (0.89–1.67)	80.4
5 mg/kg	2	0.81 (0.75–0.87)	0	2	0.89 (0.82–0.95)	0	2	2.15 (1.15–3.99)	40.7
**Chemotherapy regimen**									
Platinum	6	0.91 (0.83–1.01)	84.7	6	0.95 (0.87–1.04)	75.1	3	1.12 (0.49–2.55)	91.6
Non-platinum	4	0.78 (0.74–0.82)	0	3	0.90 (0.83–0.98)	0	4	1.51 (1.12–2.04)	28.0
**Age (years)**									
50–55	1	0.76 (0.68–0.85)	0	-	-	-	1	2.05 (1.14–3.67)	-
56–60	4	0.91 (0.78–1.07)	91.2	4	0.94 (0.81–1.08)	80.5	3	1.02 (0.80–1.38)	82.3
61–65	2	0.83 (0.79–0.87)	0	2	0.90 (0.86–0.94)	0	1	**2.64 (1.72–4.07)**	-
>66	1	0.82 (0.74–0.90)	0	1	0.95 (0.85–1.06)	-	-	-	-
**Median Follow-up (months)**									
6–12	1	0.85 (0.80–0.90)	0	1	0.91 (0.85–0.98)	-	-	-	-
13–24	3	0.76 (0.71–0.81)	0	2	0.83 (0.72–0.96)	0	2	1.53 (0.88–2.65)	51.5
25–36	5	0.86 (0.80–0.94)	58.6	5	0.92 (0.86–0.98)	35.1	3	1.12 (0.49–2.55)	91.6
>37	1	1.07 (0.99–1.16)	-	1	1.11 (1.01–1.22)	0	-	-	-
**Median duration of therapy (weeks)**									
<12	-	-	-	—	-	-	-	-	-
12–24	2	0.93 (0.70–1.23)	96.2	2	0.99 (0.79–1.24)	93.0	1	**2.64 (1.72–4.07)**	-
25–36	3	0.84 (0.74–0.94)	81.3	2	0.95 (0.90–1.00)	0	2	1.33 (0.63–2.79)	84.4
>37	1	0.76 (0.70–0.83)	-	1	0.84 (0.71–0.99)	-	1	1.29 (1.08–1.53)	-
**Time points of response assessment (weeks)**									
6	4	0.84 (0.75–0.94)	78.3	4	0.91 (0.83–1.00)	43.5	4	1.13 (0.82–1.54)	81.9
8–12	3	0.79 (0.74–0.84)	0	2	0.88 (0.82–0.94)	0	2	**2.59 (1.77–3.80)**	0
24	1	1.07 (0.99–1.16)	-	1	1.11 (1.01–1.22)	-	-	-	-
**Metastatic NSCLC**									
**BV dose**									
2.5 mg/kg	2	0.87 (0.80–0.94)	0	1	0.90 (0.69–1.18)	-	-	-	-
5 mg/kg	7	0.85 (0.81–0.89)	25.3	6	0.94 (0.90–0.99)	0	5	1.60 (1.37–87)	9.4
**Chemotherapy regimen**									
Platinum	8	0.86) 0.83–0.90)	5.3	6	0.92 (0.87–0.97)	0	3	1.57 (1.32–1.86)	18.6
Non-platinum	1	0.81 (0.75–0.88)	0	1	0.99 (0.91–1.07)	-	1	1.99 (1.17–3.37)	-
**Age (years)**									
50–55	-	-	-	-	-	-	-	-	-
56–60	3	0.89 (0.83–0.95)	20.3	1	1.07 (0.83–1.36)	-	3	1.51 (1.26–1.80)	12.8
61–65	3	0.81 (0.75–0.87)	0	3	0.98 (0.91–1.05)	0	2	1.98 (1.43–2.74)	0
>66	-	-	-	-	-	-	-	-	-
**Median Follow-up (months)**									
6–12	2	0.85 (0.74–0.97)	39.3	1	1.07 (0.83–1.36)	-	2	1.47 (1.04–2.06)	57.2
13–24	4	0.82 (0.79–0.86)	0	4	0.93 (0.88–0.98)	2.3	1	1.99 (1.17–3.37)	-
25–36	-	-	-	-	-	-	-	-	-
>37	-	-	-	-	-	-	-	-	-
**Median duration of therapy (weeks)**									
<12	-	-	-	-	-	-	-	-	-
12–24	5	0.84 (0.80–0.88)	0	4	0.92 (0.87–0.98)	0	3	1.59 (1.22–2.07)	44.4
25–36	-	-	-	-	-	-	-	-	-
>37	1	0.83 (0.65–1.06)	-	1	0.86 (0.68–1.09)	-	-	-	-
**Time points of response assessment (weeks)**									
6	6	0.82 (0.78–0.86)	0	6	0.94 (0.90–0.99)	0	3	1.64 (1.14–2.35)	52.6
8–12	1	0.88 (0.81–0.96)	-	-	-	-	1	1.69 (1.31–2.19)	-
24	-	-	-	-	-	-	-	-	-
**Metastatic Breast Cancer**									
**BV dose**									
2.5 mg/kg	2	0.91 (0.83–0.98)	0	1	1.02 (0.91–1.14)	-	2	1.18 (1.01–1.37)	0
5 mg/kg	6	0.87 (0.82–0.92)	59.8	6	0.97 (0.93–1.01)	0	7	1.42 (1.30–1.55)	0
**Chemotherapy regimen**									
Platinum	6	0.86 (0.82–0.90)	36.6	6	0.97 (0.93–1.01)	0	5	1.34 (1.20–1.49)	32.0
Non-platinum	2	0.92 (0.79–1.06)	78.9	2	0.99 (0.88–1.12)	47.5	2	1.43 (1.21–1.68)	0
**Age (years)**									
50–55	7	0.89 (0.85–0.93)	31.0	5	0.98 (0.93–1.03)	0	4	1.31 (1.19–1.44)	0
56–60	2	0.82 (0.77–0.87	11.8	2	0.96 (0.90–1.03)	0	3	1.51 (1.24–1.85)	29.5
61–65	-	-	-	-	-	-	-	-	-
>66	-	-	-	-	-	-	-	-	-
**Median Follow-up (months)**									
6–12	-	-	-	-	-	-	-	-	-
13–24	3	0.89 (0.82–0.95)	60.4	3	1.07 (0.83–1.36)	0	3	1.38 (1.22–1.55)	0
25–36	2	0.91 (0.85–0.98)	0	2	0.97 (0.93–1.00)	2.3	2	1.41 (0.93–2.12)	86.1
>37	1	0.80 (0.75–0.86)	-	1	0.93 (0.88–0.98)	-	-	-	-
**Median duration of therapy (weeks)**									
<12	**-**	-	-	**-**	-	-	**-**	**-**	**-**
12–24	-	-	-	-	-	-	-	-	-
25–36	4	0.85 (0.80–0.90)	40.5	3	0.92 (0.87–0.98)	0	4	1.46 (1.25–1.71)	15.9
>37	1	0.92 (0.83–1.01)	0	-	-	-	-	-	-
**Time points of response assessment (weeks)**									
6	2	0.94 (0.85–1.03)	53.2	2	0.99 (0.84–1.47)	28.9	2	1.42 (1.15–1.75)	0
8–12	5	0.87 (0.82–0.92)	45.9	3	0.94 (0.90–0.99)	0	4	1.38 (1.12–1.71)	62.9
24	-	-	-	-	-	-	-	-	-

**Abbreviations:** PFS: progression free survival; OS: overall survival; ORR: overall response rate; HR: hazard ratio; RR: relative risk; BV: bevacizumab; NSCLC: non-small cell lung cancer.

A statistically significant improvement in ORR was found for 61–65 years old patients compared to the other age groups in all trials combined (RR: 2.04, 95% CI: 1.67–2.49) and in the colorectal cancer trials (RR: 2.64, 95% CI: 1.72–4.07). The highest ORR was observed in studies with a median duration of therapy between 12–24 weeks for colorectal cancer (RR: 2.64, 95% CI: 1.72–4.07). The ORR was higher in colorectal cancer studies if the response was assessed between 8–12 weeks compared to 6 weeks (RR: 2.59, 95% CI: 1.77–3.80 vs. RR: 1.13, 95% CI: 0.82–1.54). For the other subgroup analyses, RR of ORR did not statistically significantly differ.

### Survival post progression

SPP calculated in 7 colorectal cancer trials was 10.6 months. In NSCLC (6 studies), breast cancer (4 studies) and ovarian cancer (3 studies) SPP times were 9.2, 15.6 and 21.1 months, respectively.

### Evaluating the relationship between PFS and OS

**Fig 5:**
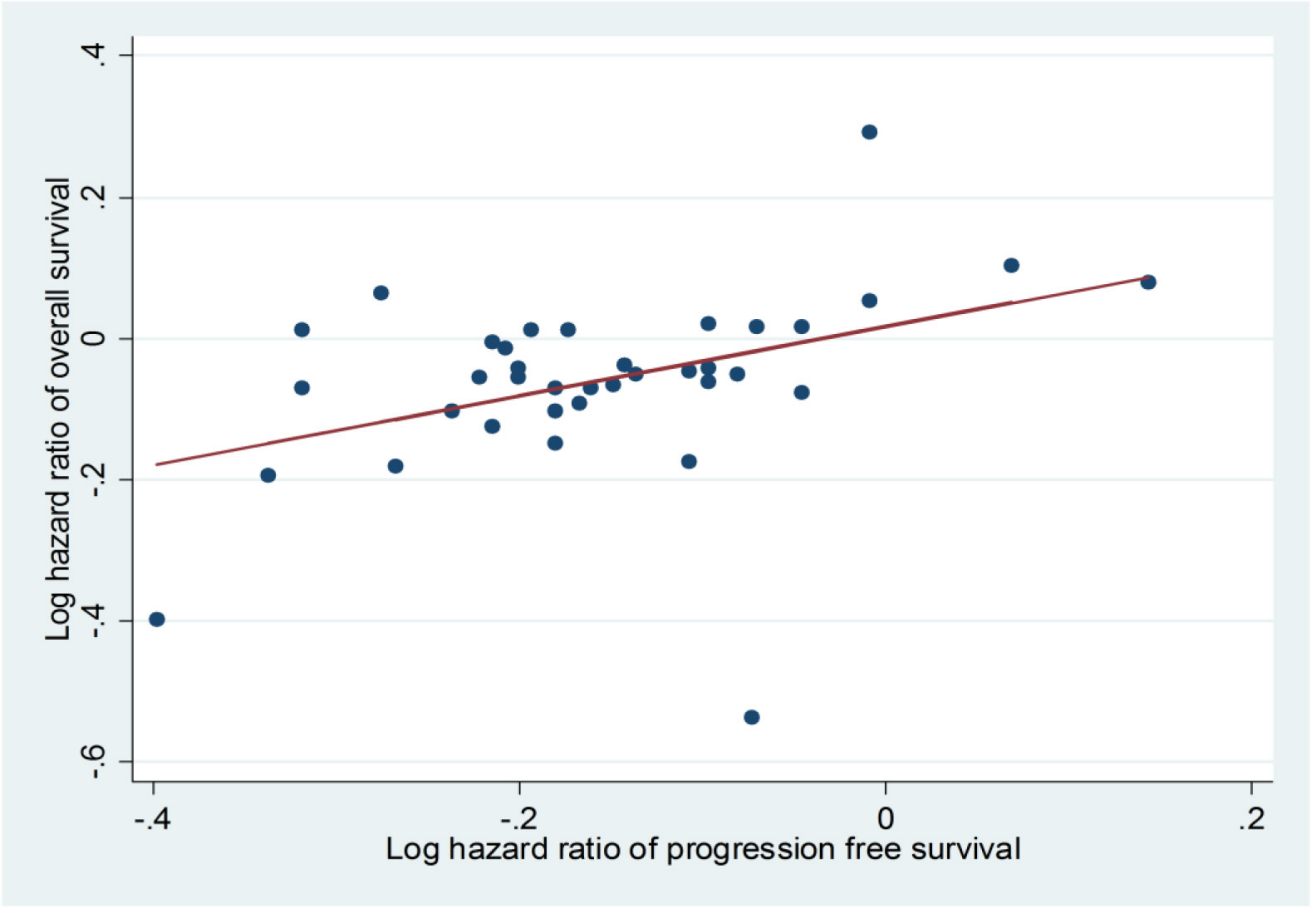
Spearman’s correlation between progression free survival and overall survival.

The results showed a significant moderate association between PFS and OS across all combined trials (The Spearman correlation coefficient (r) was 0.41, p-value: 0.01) (**Fig 5**).

Results of subgroup analyses showed that the correlation between PFS and OS was stronger in studies of metastatic breast cancers (r: 0.57; p-value: 0.18) compared to metastatic colorectal cancer (r: 0.40; p-value: 0.24) and NSCLC (r:-0.45; p-value: 0.31).

### Publication bias

**Fig 6:**
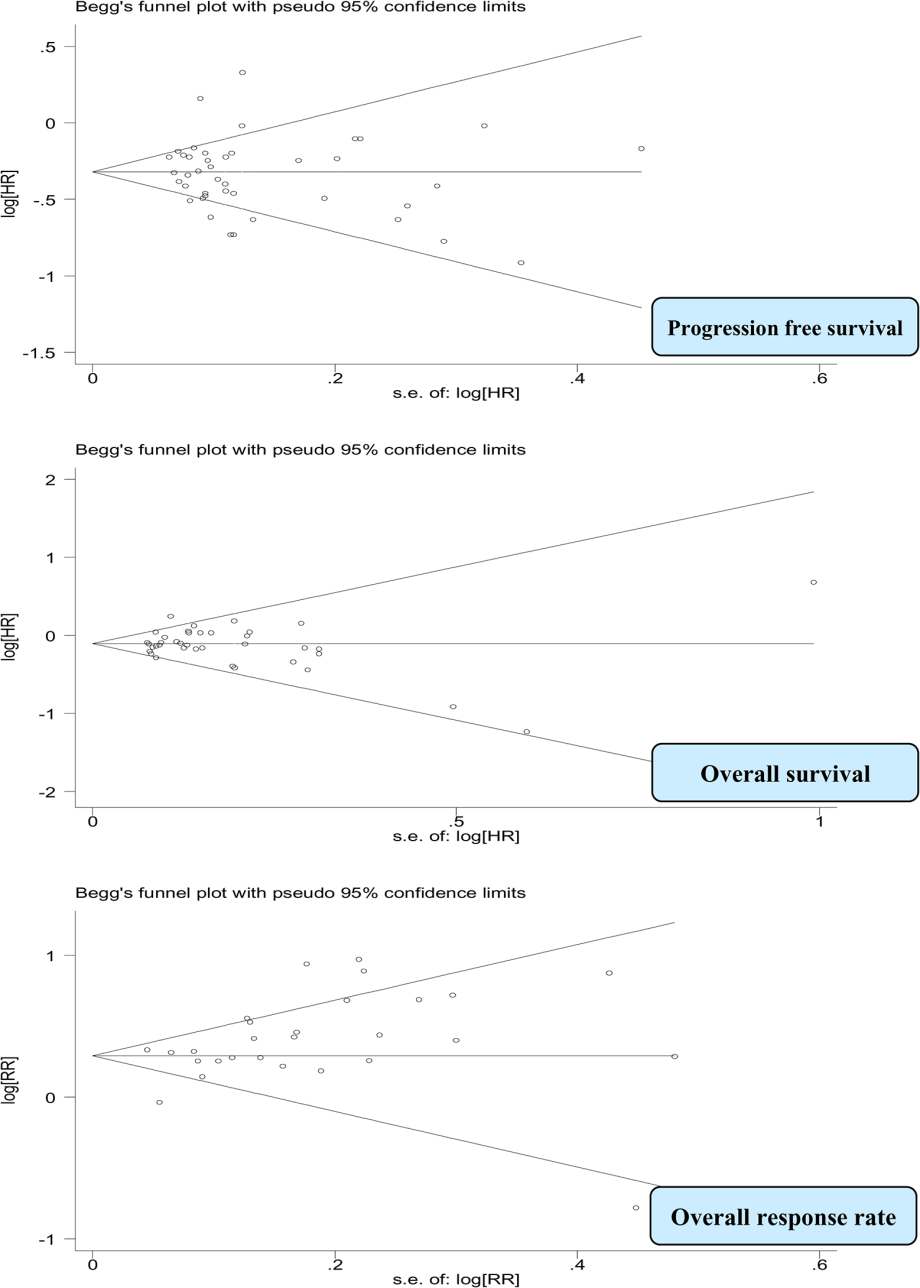
Funnel plots for efficacy assessment in all trials combined.

The funnel plots did not show evidence of significant publication bias for PFS and OS (p-values: 0.42, 0.69, respectively). However for ORR, the funnel plot appeared to be asymmetric, and there was evidence of bias using the Egger (weighted regression) method (P for bias was 0.02). It appeared that small trials producing more pronounced effects were missing **(Fig 6)**.

### Heterogeneity

Moderate heterogeneity was observed for PFS (I^2^: 72.4%) and for ORR (I^2^: 71.3%) in all combined trials **(Table 2)**. We further explored the causes of the heterogeneity in different tumor types. PFS and ORR were more heterogeneous when analyzed separately in colorectal cancer trials (I^2^: 82.3% and 84.2%, respectively). Other stratified subgroup analyses were performed and indicated the large differences in the HRs of PFS and RRs of ORR across BV dose and adjuvant therapy agents in colorectal cancer trials **(Tables 3&4)**.

### Meta-regression analysis

The potential influence of patient and trial characteristics including BV dose, participant’s age, median duration of follow-up and median duration of BV therapy on study outcomes was explored in meta-regression analyses. The analyses showed that none of these characteristics statistically significantly influenced PFS and OS in all trials combined. However BV dose was found to be a predictor of ORR benefit (p-value: 0.02) (**Table 5**).

**Table 5:** Meta-regression analyses.

	Predictors p-value			
	BV dosage	Patient’s age	Median duration of follow-up	Median duration of BV therapy
**PFS**	0.48	0.05	0.53	0.87
**OS**	0.33	0.18	0.71	0.15
**ORR**	**0.02**	0.20	0.48	1.00

**Abbreviations:** PFS: progression free survival; OS: overall survival; ORR: overall response rate; BV: bevacizumab.

### ADRs analyses

ADRs data was available on all grades and on those ADRs with grade 3 (severe) or more (life threatening). ADRs were reported differently among 44 RCTs **(Table 6)**. BV was associated with a higher risk of all grade ADRs e.g. thrombocytopenia, hypertension, bleeding and thromboembolic events. A higher risk of severe grade events such as wound healing complication, epistaxis and stomatitis was also observed in patients treated by BV. The highest risk was found for severe grade hypertension (RR: 5.83, 95% CI: 4.44–7.65) which was reported in 40 trials where 1,149 patients out of 16,437 in the BV treated group and 147 patients out of 15,378 in the control group were diagnosed with this ADR. BV significantly reduced the risk of both all grade (RR: 0.83, 95% CI: 0.71–0.98) and severe grade (RR: 0.78, 95% CI: 0.66–0.93) anemia as well as severe grade fatigue (RR: 0.58, 95% CI: 0.38–0.87) compared with adjuvant therapy alone in cancer patients. No statistically significant differences between patients with and without BV in their regime were found for venous thromboembolic events, fistula abdominal abscess, leukopenia, cardiac events including left ventricular (LV) dysfunction and congestive heart failure and pulmonary events including embolism, dyspnea, pneumonitis and hemorrhages.

**Table 6:** Safety assessment of ADRs with BV in all trials combined.

	All grade				Severe grade			
ADRs	No. of studies included	No. of patients in BV group/sample size	No. of patients in control group/sample size	RR (95% CI)	No. of studies included	No. of patients in BV group/sample size	No. of patients in control group/sample size	RR (95% CI)
**Venous Thromboembolic Events**	6	156/10621	127/9977	1.16 (0.82–1.64)	19	507/10621	410/9977	1.18 (0.98–1.43)
**Thrombocytopenia**	8	192/6755	148/6537	1.23 (1.01–1.49)	21	365/6755	323/6537	1.13 (0.89–1.44)
**Anemia**	5	221/6955	257/6744	0.83 (0.71–0.98)	17	236/6955	295/6744	0.78 (0.66–0.93)
**Hypertension**	13	723/16437	190/15378	3.46 (2.72–4.41)	40	1149/16437	147/15378	5.83 (4.44–7.65)
**Bleeding**	7	310/14173	114/13269	2.71 (1.80–4.09)	30	276/14173	139/13269	1.84 (1.43–2.35)
**Thromboembolic Events**	4	113/4560	82/4486	1.35 (1.04–1.76)	11	161/4560	96/4486	1.81 (1.10–2.97)
**Arterial Thromboembolic Events**	7	48/7775	24/7162	1.49 (0.90–2.45)	14	99/7775	51/7162	1.68 (1.13–2.50)
**Gastrointestinal perforation**	7	41/11677	16/11094	1.97 (1.07–3.64)	23	201/11677	124/11094	2.06 (1.27–3.34)
**Wound Healing Complication**	3	13/7312	6/7155	1.90 (0.74–4.88)	12	51/7312	21/7155	1.94 (1.08–3.49)
**Fistula Abdominal Abscess**	3	9/5199	1/5092	4.51 (0.97–20.91)	8	38/5199	21/5092	1.51 (0.51–4.42)
**Neutropenia**	8	339/13519	259/12492	1.20 (1.05–1.37)	32	3913/13519	3606/12492	1.06 (1.01–1.12)
**Febrile Neutropenia**	5	189/9940	104/9208	1.55 (1.09–2.19)	20	383/9940	247/9208	1.42 (1.22–1.66)
**Leukopenia**	4	84/3810	65/3692	1.26 (0.94–1.68)	10	411/3810	183/3692	1.52 (0.98–2.36)
**Proteinuria**	8	524/13562	142/12621	4.98 (2.11–11.71)	30	299/13562	36/12621	4.90 (3.53–6.80)
**Diarrhea**	12	533/7807	476/7600	1.09 (0.97–1.22)	23	683/7807	533/7600	1.21 (1.07–1.37)
**Epistaxis**	4	154/977	46/989	3.23 (2.38–4.38)	6	14/977	3/989	3.84 (1.29–11.37)
**Stomatitis**	4	133/2910	69/2742	1.87 (1.45–2.39)	6	97/2910	28/2742	3.25 (2.14–4.93)
**Vomiting**	7	28/6787	16/6540	1.04 (0.87–1.24)	17	3/6787	2/6540	1.33 (1.00–1.76)
**Nausea**	8	579/6703	561/6502	1.02 (0.93–1.11)	18	225/6703	151/6502	1.42 (1.13–1.78)
**Fatigue**	7	766/6518	727/6329	1.01 (0.93–1.10)	7	566/6518	413/6329	0.58 (0.38–0.87)
**Rash**	4	196/1815	157/1823	1.50 (0.93–2.43)	6	87/1815	33/1823	2.49 (1.69–3.66)
**Cardiac events** *	6	106/9070	75/8560	1.20 (0.89–1.61)	16	95/9070	72/8560	1.27 (0.88–1.83)
**Pulmonary events** **	3	18/2214	25/2177	0.72 (0.40–1.31)	6	24/2214	31/2177	0.77 (0.45–1.32)

**Abbreviation:** ADRs: adverse drug reactions; RR: relative risk; BV: bevacizumab.

*Cardiac events including: left ventricular (LV) dysfunction and congestive heart failure.

**Pulmonary events including: embolism, dyspnea, pneumonitis and hemorrhages.

### ADRs and BV dose

We assessed whether the higher dose of BV is related to the risk for developing ADRs in cancer patients **(Table 7)**. When comparing the risk of ADRs between low and high-doses of BV, the RRs of all grade proteinuria (2.64; 95% CI: 1.29–5.40 vs. 9.24; 95% CI: 6.60–12.94) and severe grade bleeding (1.36; 95% CI: 1.05–1.75 vs. 2.87; 95% CI: 1.97–4.18) was increased significantly when switching from 2.5 mg/kg to 5 mg/kg BV. Although not statistically significant, there was a trend towards a higher risk of several side effects (including all grade and severe grade of hypertension, gastrointestinal perforation, thrombocytopenia, diarrhea, neutropenia and febrile neutropenia, all grade epistaxis and severe grade of ADRs including rash, nausea, vomiting, arterial thromboembolic events and cardiac events) in patients using high-dose compared with low-dose BV.

**Table 7:** Safety subgroup analyses in all trials combined.

	All grade		Severe grade	
	RR (95% CI) by BV dosage (mg/kg)		RR (95% CI) by BV dosage (mg/kg)	
	2.5 (mg/kg)	5 (mg/kg)	2.5 (mg/kg)	5 (mg/kg)
**Hypertension**	2.66 (2.15–3.29)	4.71 (3.10–7.15)	4.47 (3.05–6.56)	7.48 (5.04–11.10)
**Bleeding**	2.35 (1.54–3.60)	3.53 (2.43–5.13)	**1.36 (1.05–1.75)**	**2.87 (1.97–4.18)**
**Gastrointestinal perforation**	2.07 (0.24–18.04)	2.18 (1.12–4.22)	1.86 (0.98–3.55)	2.44 (1.18–5.04)
**Proteinuria**	**2.64 (1.29–5.40)**	**9.24 (6.60–12.94)**	4.18 (2.67–6.55)	6.42 (3.66–11.26)
**Epistaxis**	2.95 (2.10–4.15)	4.60 (2.35–8.99)	4.85 (0.23–10.56)	3.71 (1.16–11.86)
**Stomatitis**	1.91 (1.47–2.48)	1.54 (0.69–3.44)	3.62 (0.88–14.84)	3.22 (2.08–4.97)
**Rash**	1.80 (0.87–3.72)	1.24 (0.48–3.20)	1.80 (0.69–4.59)	2.77 (1.72–4.45)
**Neutropenia**	1.16 (0.89–1.53)	1.22 (1.04–1.44)	1.03 (0.93–1.14)	1.09 (1.03–1.44)
**Febrile neutropenia**	1.32 (0.30–5.82)	1.52 (1.01–2.30)	1.37 (1.01–1.85)	1.45 (1.21–1.73)
**Fatigue**	0.93 (0.78–1.11)	1.05 (0.95–1.17)	0.47 (0.27–0.84)	0.68 (0.33–1.37)
**Nausea**	1.04 (0.88–1.22)	0.98 (0.87–1.11)	1.19 (0.83–1.71)	1.82 (1.33–2.50)
**Vomiting**	1.07 (0.88–1.30)	0.84 (0.47–1.50)	1.15 (0.83–1.58)	1.69 (1.04–2.74)
**Thrombocytopenia**	1.16 (0.88–1.54)	1.40 (0.96–2.02)	0.94 (0.63–1.40)	1.32 (1.00–1.74)
**Arterial thromboembolic events**	1.91 (0.63–5.79)	1.42 (0.72–2.79)	1.50 (0.98–2.29)	2.78 (1.13–6.85)
**Diarrhea**	1.04 (0.96–1.14)	1.46 (0.89–2.40)	1.21 (1.03–1.42)	1.23 (0.91–1.67)
**Cardiac events** *	1.33 (0.19–9.55)	1.19 (0.89–1.61)	0.97 (0.48–1.95)	1.45 (0.93–2.27)
**Pulmonary events** **	0.72 (0.38–1.38)	0.72 (0.16–3.19)	0.86 (0.36–1.99)	0.74 (0.27–1.99)

**Abbreviation:** RR: relative risk; BV: bevacizumab.

*Cardiac events including: left ventricular (LV) dysfunction and congestive heart failure.

**Pulmonary events including: embolism, dyspnea, pneumonitis and hemorrhages.

### Sensitivity analysis

We performed a sensitivity analysis for all grade and severe grade hypertension and proteinuria excluding the 2 trials [22, 23] concerning patients with renal cancer. The magnitude of association was lower after excluding these trials, but remained robustly significant: for all grade and severe grade hypertension (3.06, 95%CI: 2.47–3.79 and 5.72, 95%CI: 4.35–7.51, respectively) and for all grade (3.12, 95%CI: 1.59–6.13) and severe grade proteinuria (4.43, 95%CI: 3.17–6.20).

### QOL

QOL was assessed in 7 RCTs [19,31,36,41,47,48,50] at baseline and during follow-up until disease progression. We were not able to conduct a meta-analysis for this outcome because QOL was measured using different instruments in different trials. All trials reported that there were no statistically significant differences in the mean change in QOL between patients treated by BV compared to patients treated with chemotherapeutic agents alone.

## Discussion

To the best of our knowledge, this is the largest meta-analysis of BV that evaluated both efficacy and safety in different types of solid tumors in cancer patients. Compared to previous published meta-analyses, our study adds relevant information with respect to the identification of predictors of BV risk and benefit through subgroup and meta-regression analyses. This meta-analysis confirmed that the addition of BV to adjuvant therapy leads to improvement in PFS, OS and ORR in all trials combined. This PFS and OS improvement was observed across various tumor types and BV doses, and across different patient or trial characteristics. In contrast, ORR appeared to be influenced by tumor type and BV dose. Despite increased risk of expected ADRs, the addition of BV to adjuvant therapy does not seem to influence QOL. Though not statistically significant, there was a trend towards a higher risk of several ADRs in patients using high-dose BV compared to patients treated with low-dose.

The quality of each included RCT was assessed by applying the Cochrane Collaboration’s tool, which is a validated assessment instrument. The results showed a high risk of bias in domain of blinding for both primary and secondary outcomes, however because in cancer trials most outcomes (like OS and PFS) are not likely to be influenced by lack of blinding the overall quality of all trials combined was considered to be acceptable [52]. Lack of blinding might have influenced the results of the QOL analyses, and therefore such results should be interpreted with caution.

We demonstrated improvement in PFS in patients with all types of tumors except for patients with melanoma, mesothelioma or cervical cancers. In spite of OS improvement when all trials were combined, no significant OS advantage was observed in certain types of cancer e.g. breast cancer and ovarian cancer although there were trends in the correct direction. Even after adding several new trials to the meta-analyses our findings continued to show this lack of OS benefit in breast cancer [4, 53] and ovarian cancer [54]. A possible explanation according to Broglio et al [55] is that when SPP is long, for example 15.6 months for breast cancer and 21.1 months for ovarian cancer, it is more difficult to show improvement in OS. While in trials with a short SPP such as for colorectal cancer (10.6 months) and NSCLC (9.2 months) there is usually a statistically significant benefit in OS if there is a statistically significant treatment benefit in PFS.

On the other hand, surrogacy of PFS for OS in different cancer types has been evaluated in several studies with different results [56, 57]. In the current study, a significant moderate correlation between PFS and OS was observed in all combined trials which is consistent with the results of previous studies. Therefore, in clinical trials with a PFS benefit, lack of statistical significance in OS does not necessarily mean a lack of improvement in OS.

Our study also suggests that the relationship between PFS and OS varies considerably by cancer type. The correlation between PFS and OS was more pronounced in trials of breast cancer compared with colorectal cancer and in NSCLC there was a surprisingly negative correlation. In breast cancer and colorectal cancer, our results are in line with the results of previous studies [58, 59] while in NSCLC there is evidence for a positive relationship between PFS and OS [60].Most malignant tumors are highly dependent on angiogenesis, therefore it is as expected that BV added to standard chemotherapies substantially improves the ORR in different tumor types.

The dose of BV used in adjuvant therapy was not found to be associated with PFS or OS benefit, consistent with a prior meta-analysis [4]. However, our findings showed that the ORR benefit varied significantly by tumor type. The highest ORR was observed in renal cancer patients, while gastric cancer patients benefited the least of BV treatment. Thus, tumor type likely plays an important role in the response to BV. This variation in response may also be partly due to the combination of BV with different chemotherapeutic agents in different tumor types. However, other reasons might be differences in the number of studies and power of the studies for the different tumor types. Further research is warranted into which tumors benefit most from BV therapy.

Although not significant, there was a trend towards a higher ORR in patients using a high-dose BV compared with patients using a low-dose in all trials combined, in colorectal cancer and in breast cancer trials. The increase in ORR with high-dose BV may have been due to improved BV-induced drug delivery to the tumor site. Our results are in line with the results of previous studies that showed a dose-response relationship in NSCLC and metastatic renal cell carcinoma [61, 62], but not in colorectal cancer [14]. A significantly higher improvement in ORR found in 61–65 years old patients compared with all other age groups in all trials combined and in colorectal cancer trials has no biological or clinical explanation, and is likely a chance finding. An important consideration is the timing of response assessment: the results in the colorectal cancer studies showed a higher ORR in favor of BV if response was assessed between 8–12 weeks compared with 6 weeks. A longer time to response assessment is likely to capture slower tumor responses and be more complete, important for non-cytotoxic agents such as BV.

Our results showed that some ADRs were more common in patients randomized to BV. This is consistent with those of prior safety meta-analyses linking specific ADRs to BV therapy [3,53,63–71]. BV significantly reduced the risk of both all grade and severe grade anemia compared with adjuvant therapy alone in cancer patients with no significant variation among different BV doses which was in line with previous meta-analysis [65]. Several possibilities related to VEGF inhibition may explain the effect of BV on anemia. BV has been shown to promote hepatic erythropoietin (EPO) synthesis and erythrocytosis in preclinical models [72]; also, it may cause tissue hypoxia due to its anti-angiogenesis and vasoconstriction effect, leading to subsequent up-regulation of erythropoietin. Furthermore, we showed that the addition of BV to standard adjuvant therapy was associated with reduced risk of severe grade fatigue in cancer patients compared to those who were treated by adjuvant therapy alone. Increase in several inflammatory markers is associated with an increase in fatigue among cancer patients during and after cancer treatment [73], so BV may reduce these inflammatory markers via an unknown mechanism; alternatively, this may be associated with a reduction in anemia. Since this finding has not been reported before it might be interesting for future research.

Our study adds information to existing literature about the increased risk for the following ADRs: febrile neutropenia, stomatitis, vomiting, nausea and rash as well as decreased risk for severe grade fatigue.

In our study, the most frequent ADRs of BV was hypertension which represents a common finding across 40 trials. Infusion of VEGF has been found to produce hypotension [74] and thus blockade of VEGF may potentially lead to elevation of blood pressure.

We also investigated the associations of BV with ADRs according to different BV dose. Our study indicated a dose dependency although not significantly for the association of most ADRs with BV therapy. Moreover, a significant higher risk of all-grade proteinuria and severe grade bleeding was observed in patients who received high-dose compared with patients treated with low-dose BV (RR: 9.24 vs. 2.64) and (2.87 vs. 1.36), respectively which is in agreement with previous meta-analyses [1,2].

In this study, the following limitations were acknowledged: This study was conducted using published RCTs not individual patient data. Our meta-analysis pooled trials with heterogeneous cancer types and different patient populations, BV doses, antineoplastic agents used, follow-up durations and the timing of response assessment, although by applying a random-effects model we took possible heterogeneity into account. In this meta-analysis we have decided not to include studies on glioblastoma because in this kind of tumor, drug delivery is different from the other types of cancer that were studied. Furthermore, we only compared the efficacy and safety of BV with standard chemotherapy agents not with radiotherapy and other types of anti-neoplasms.

As in all meta-analyses, our results may be biased as a result of potential publication bias however; a funnel plot evaluation for the primary endpoints did not indicate serious publication bias. Finally, most of trials did not report outcomes separately by patient’s gender, so we were not being able to perform a subgroup analysis to evaluate a potential gender treatment interaction.

In summary, this meta-analysis extends the results of previous RCTs and meta-analyses which show a benefit of adding BV to adjuvant therapy compared to patients who received adjuvant therapy alone, but finding no interaction with baseline characteristics or with dose of BV. In contrast, patients treated with high-dose BV had more ADRs but without significant changes in measured QOL. Future studies are needed to evaluate the efficacy and safety of the addition of other angiogenesis inhibitors to standard chemotherapy agents and to compare these results with the effect of BV on PFS and OS.

## Supporting Information

S1 PRISMA Checklist PRISMA checklist. (PDF)

S1 File Meta-analysis of the log hazard ratios of progression free survival comparing bevacizumab and standard chemotherapy in Figure A: metastatic colorectal cancer, Figure B: metastatic non-small cell lung cancer (NSCLC) and Figure C: metastatic breast cancer. (TIF)

S2 File Meta-analysis of the log hazard ratios of overall survival comparing bevacizumab and standard chemotherapy in Figure A: metastatic colorectal cancer, Figure B: metastatic non-small cell lung cancer (NSCLC) and Figure C: metastatic breast cancer. (TIF)

S3 File Meta-analysis of the risk ratios of overall response rate comparing bevacizumab and standard chemotherapy in Figure A: metastatic colorectal cancer, Figure B: metastatic non-small cell lung cancer (NSCLC) and Figure C: metastatic breast cancer. (TIF)

S1 Table Search strategy. (PDF)

S2 Table Risk of bias assessment in all included trials. (PDF)
