# Correction: Efficacy and Safety Assessment of the Addition of Bevacizumab to Adjuvant Therapy Agents in Cancer Patients: A Systematic Review and Meta-Analysis of Randomized Controlled Trials

**DOI:** 10.1371/journal.pone.0141704

**Published:** 2015-10-22

**Authors:** Fariba Ahmadizar, N. Charlotte Onland-Moret, Anthonius de Boer, Geoffrey Liu, Anke H. Maitland-van der Zee

The phrases “log hazard ratio” and “log HR” appear incorrectly throughout the article and Supporting Information files, with the exception of the “Data analysis” subsection, Fig 5, and Fig 6. The correct terminology in all other cases should be “hazard ratio” and “HR.”
