# Supplementary figures and images for: Efficacy and Safety Assessment of the Addition of Bevacizumab to Adjuvant Therapy Agents in Cancer Patients: A Systematic Review and Meta-Analysis of Randomized Controlled Trials
Source: PLoS One. 2015 Sep 2;10(9):e0136324. doi: 10.1371/journal.pone.0136324 (PMC4558033; doi:10.1371/journal.pone.0136324)

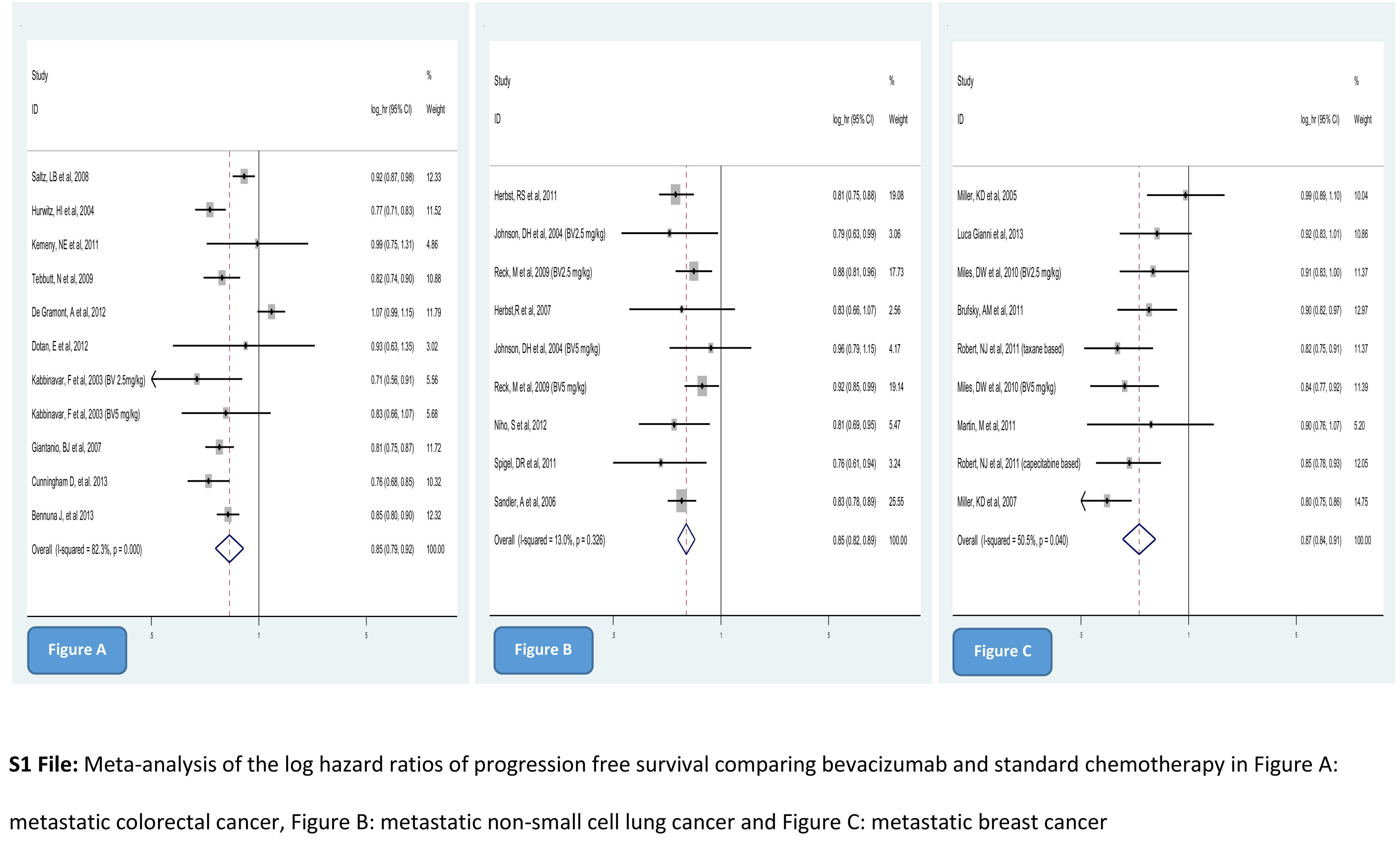

Supplement: S1 File — (TIF) [file pone.0136324.s002.tif]

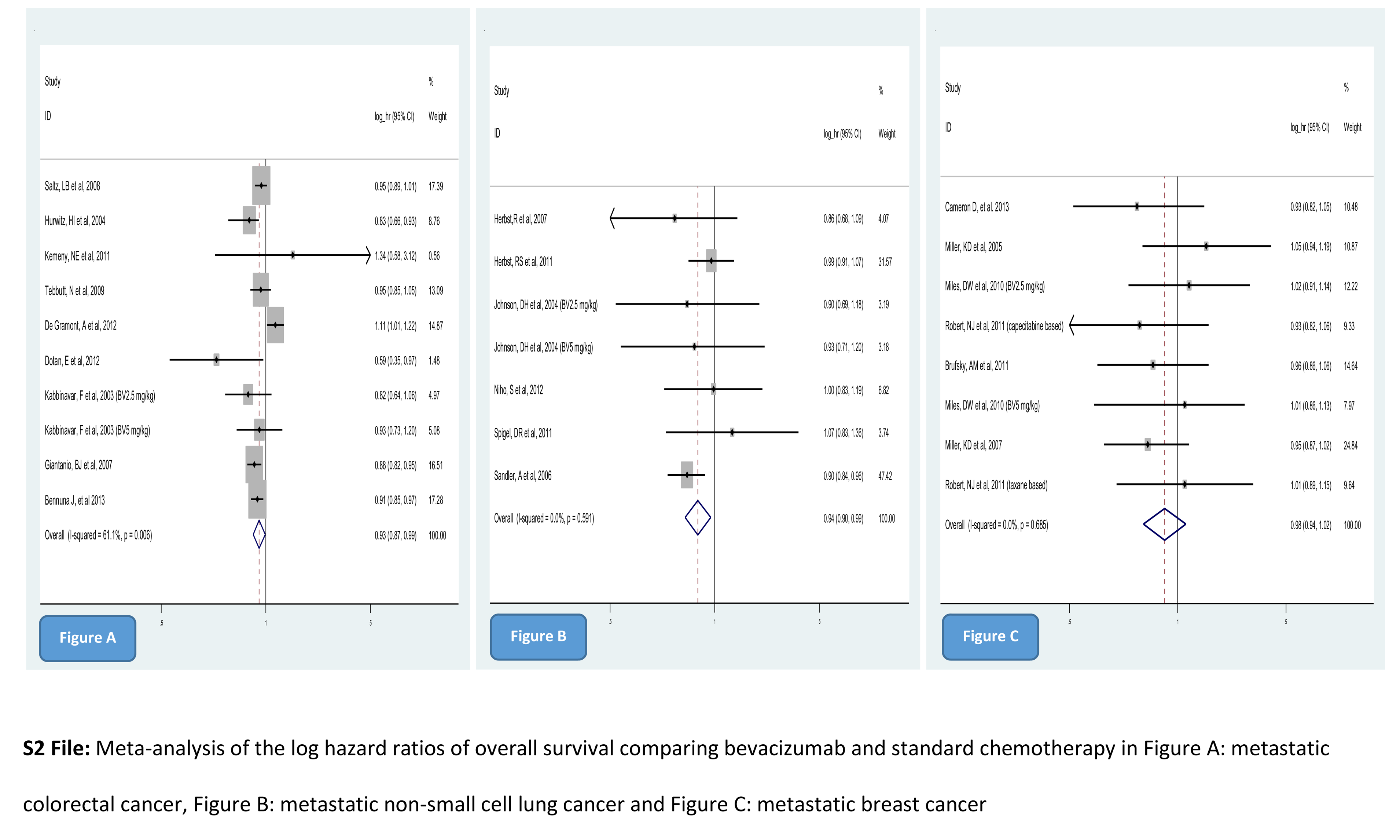

Supplement: S2 File — (TIF) [file pone.0136324.s003.tif]

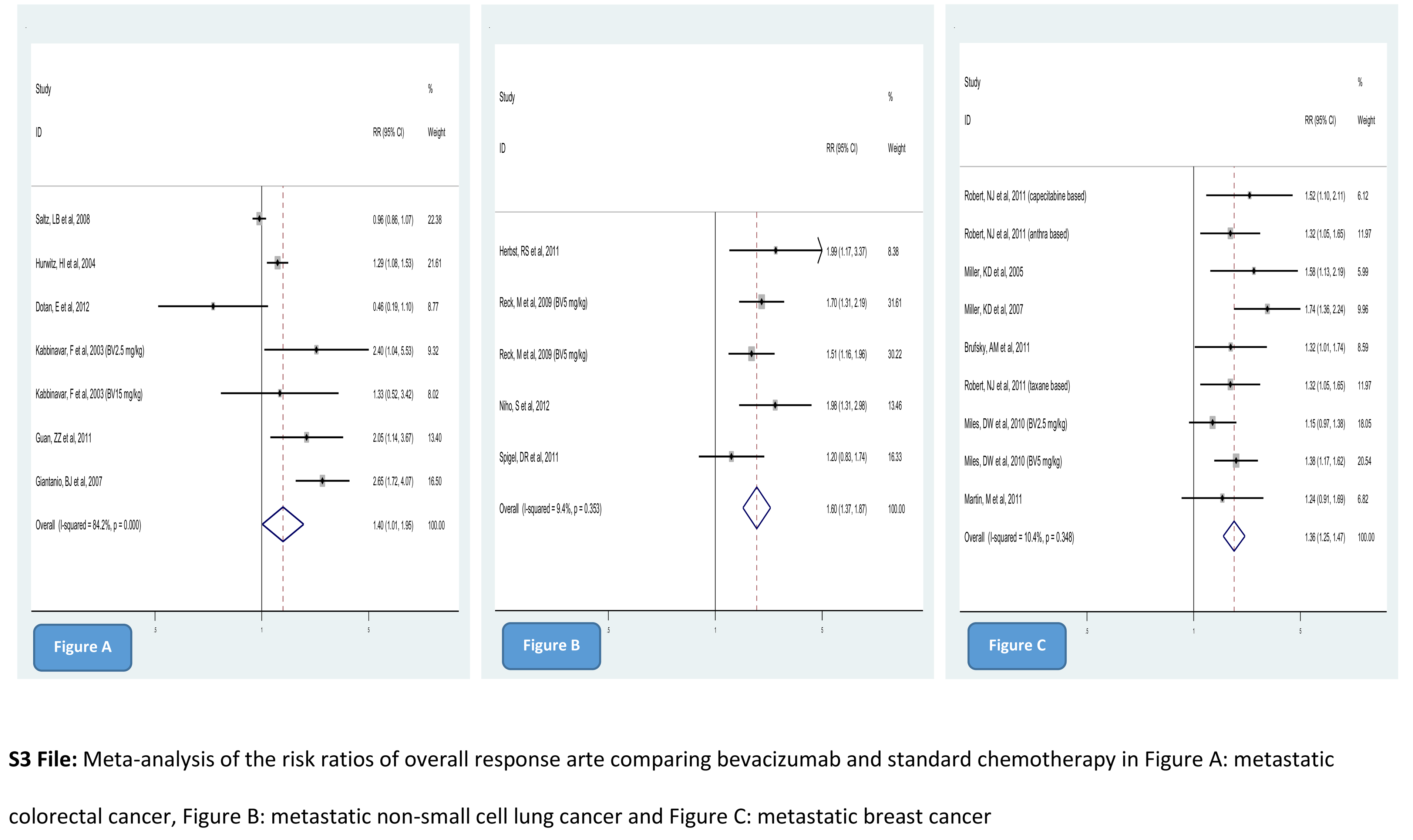

Supplement: S3 File — (TIF) [file pone.0136324.s004.tif]
