# Supplementary material for: Efficacy and Safety Assessment of the Addition of Bevacizumab to Adjuvant Therapy Agents in Cancer Patients: A Systematic Review and Meta-Analysis of Randomized Controlled Trials
Source: PLoS One. 2015 Sep 2;10(9):e0136324. doi: 10.1371/journal.pone.0136324 (PMC4558033; doi:10.1371/journal.pone.0136324)
Supplement: S1 Table — (PDF) [file pone.0136324.s005.pdf]

S1 Table: Search strategy

Design		Determinant (s)		Outcome (s)		
Randomized controlled trial	AND	Bevacizumab	AND	Safety	OR	Efficacy
Randomized clinical trial	AND	Avastin	AND	Toxicity	OR	Benefit
Randomized trial	AND			Adverse drug reaction	OR	Effectiveness
				Adverse drug effect	OR	
				Adverse drug event	OR	
				Side effect	OR	
				Risk	OR	
