# Supplementary material for: Efficacy and Safety Assessment of the Addition of Bevacizumab to Adjuvant Therapy Agents in Cancer Patients: A Systematic Review and Meta-Analysis of Randomized Controlled Trials
Source: PLoS One. 2015 Sep 2;10(9):e0136324. doi: 10.1371/journal.pone.0136324 (PMC4558033; doi:10.1371/journal.pone.0136324)
Supplement: S2 Table — (PDF) [file pone.0136324.s006.pdf]

S3 Table: Risk of bias assessment in all included trials

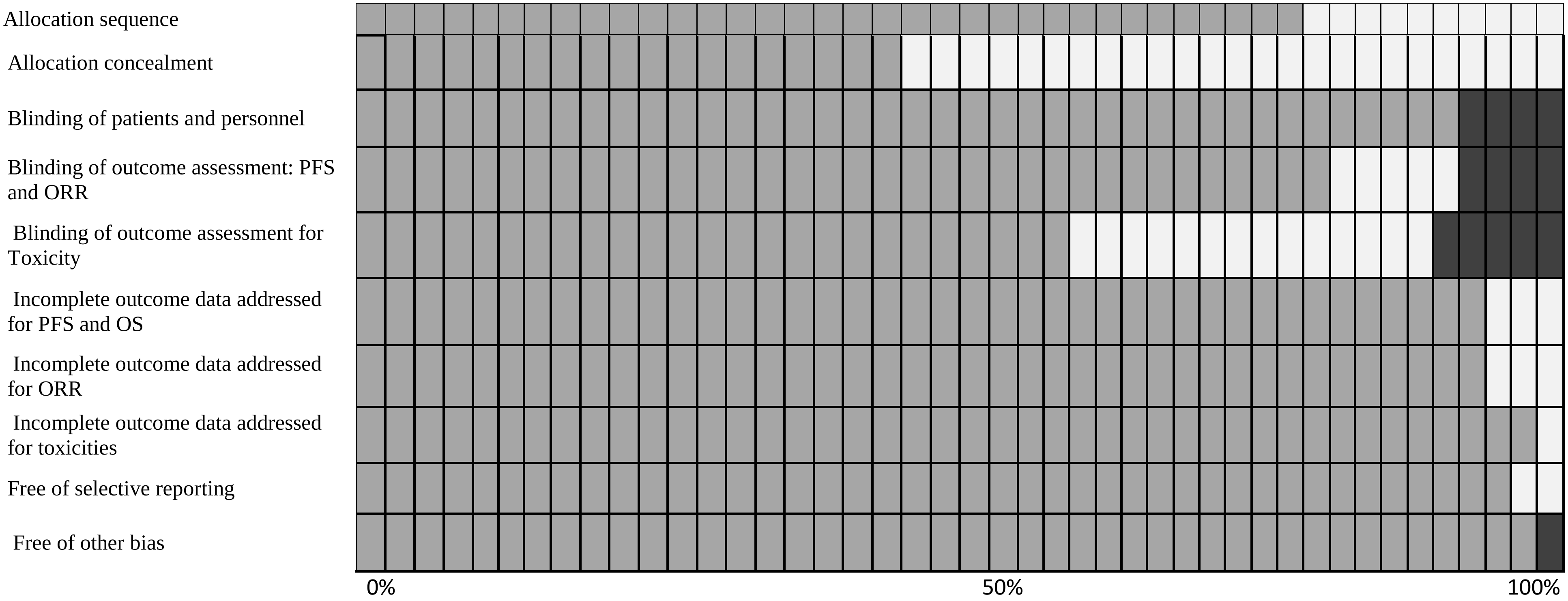

Abbreviation: PFS, progression free survival; OS, overall survival; ORR, overall response rate

Yes (low risk of bias)

Unclear (Medium risk of bias)

No (high risk of bias)
